# SmartMom Text Messaging for Prenatal Education: A Qualitative Focus Group Study to Explore Canadian Women’s Perceptions

**DOI:** 10.2196/publichealth.6949

**Published:** 2017-02-07

**Authors:** Sarah Munro, Amber Hui, Vanessa Salmons, Carolyn Solomon, Emily Gemmell, Nahal Torabi, Patricia A Janssen

**Affiliations:** ^1^ Department of Family Practice Faculty of Medicine University of British Columbia Vancouver, BC Canada; ^2^ The Dartmouth Institute for Health Policy and Clinical Practice Geisel School of Medicine Dartmouth College Hanover, NH United States; ^3^ School of Population and Public Health Faculty of Medicine University of British Columbia Vancouver, BC Canada; ^4^ Maternal, Infant, Child and Youth, Public Health Northern Health Authority Quesnel, BC Canada; ^5^ Maternal and Women’s Health Public Health Services Branch, Population and Public Health Division British Columbia Ministry of Health Victoria, BC Canada; ^6^ Faculty of Health Sciences Simon Fraser University Burnaby, BC Canada

**Keywords:** pregnancy, text messaging, prenatal education, health behavior

## Abstract

**Background:**

We engaged Canadian women in the development of a prenatal education program delivered via one-way text messaging called SmartMom. SmartMom is the first peer-reviewed, evidence-based mHealth program for prenatal education in Canada and the first to be endorsed by the Society of Obstetricians and Gynaecologists of Canada.

**Objective:**

To explore women’s preferences for a prenatal education program by text messaging.

**Methods:**

We conducted a qualitative focus group study in three Canadian communities in the Northern Health Authority. Women completed a demographic questionnaire, participated in a guided discussion about their pregnancy information-seeking behavior, reviewed a printed copy of the SmartMom text messages, and then engaged in a moderated discussion about their perceptions of the usability of the SmartMom program. Open-ended questions explored women’s perceptions regarding the message content, acceptability of receiving information by text message, positive health behaviors they might engage in after receiving a message, modifiable program factors, and intention to use the program. Thematic analysis of transcribed audio recordings was undertaken and modifications were made to the SmartMom program based on these findings.

**Results:**

A total of 40 women participated in seven focus groups in three rural northern communities. The vast majority had a mobile phone (39/40, 98%), used text messages “all the time” (28/40, 70%), and surfed the Internet on their phone (37/40, 93%). Participants perceived SmartMom to be highly acceptable and relevant. The text message modality reflected how participants currently sought pregnancy-related information and provided them with local information tailored to their gestational age, which they had not received through other pregnancy resources. Women recommended adding the opportunity to receive supplemental streams of messages tailored to their individual needs, for example, depression, pregnancy after previous cesarean, >35 years of age, new immigrants, and harm reduction for smoking and alcohol.

**Conclusions:**

This formative qualitative evaluation provides evidence that a prenatal education program by text messaging, SmartMom, is acceptable to the end users. These findings support the usability of the SmartMom program at a population level and the development of an evaluation program exploring the effects of the text messages on adoption of health-promoting behaviors and maternal-child health outcomes.

## Introduction

Prenatal education is designed to teach women and their support persons about the physiologic and psychological changes of pregnancy; what to expect during prenatal care; and how to prepare for labor, birth, and newborn care [1]. A Cochrane systematic review conducted by Gagnon and Sandall in 2007 [2], currently being updated by Brixval et al [3], investigated the effect of structured individual or group antenatal education and concluded that the effect of general antenatal education on both health and psychosocial outcomes is unclear. A more recent systematic review of the international literature investigating the effect of antenatal education on labor and birth found that positive effects might include fewer false labor admissions, less anxiety, and more partner involvement [4]. In Canada, the first national study of women’s childbirth experiences reported that 65.6% of Canadian women attended prenatal classes in their first pregnancies [5]. However, women living in a household at or below the low-income cutoff were less likely to attend classes (24.1%, 95% CI 21.6-26.7) than women living in a household above the low-income cutoff (34.7%, 95% CI 33.5-35.9) [5]. In Canadian rural and remote settings, access is a significant issue and prenatal classes may be offered as infrequently as once per year and require travel to other communities [6].

Internationally, women have significant gaps in understanding the determinants of healthy pregnancy outcomes, such as the role of healthy weight gain [7] and nutrition [8]. In Canada, an Ontario population-based survey with a 94% response rate indicated that only 25% of respondents had been told by their caregiver that there were risks associated with inappropriate weight gain [9]. A 2009 study in Toronto reported that 80% of pregnant women believed that an influenza vaccine given during pregnancy caused birth defects [10]. Typically, prenatal appointments with family doctors and obstetricians last only 10-15 minutes and thus physicians tend to focus on biomedical issues rather than on health promotion and education [11]. Consequently, there is a need for resources in an accessible and acceptable format that provide pregnant women and families with the information and skills to engage in healthy behaviors during pregnancy, labor, and birth.

An emerging body of evidence supports the utility of text messaging in provoking attitudinal and behavior change in the areas of smoking cessation, alcohol consumption, and disease management [12-14]. Short message service (SMS) text messaging interventions combine the reach and scalability of mass media with the personalization of health communication through the tailoring of messages to individuals’ health needs and preferences [15,16]. Mobile phone messaging is also useful in accessing remote or hard-to-reach populations and in providing patients with timely health information and education [15].

In the United States, the Text4baby program was launched in 2010 to provide prenatal education via text message to reduce the rate of premature birth and infant death, particularly among low-income and minority groups. As of December 2016, the service had enrolled over 1,077,628 English- and Spanish-speaking women since launching [16,17]. A randomized controlled trial of the Text4baby program among female military health beneficiaries in 2013 found that high exposure to Text4baby had a significant effect on self-reported alcohol consumption postpartum (odds ratio [OR] 0.212, 95% CI 0.046-0.973) [14]. Exposure to at least one message on prenatal health vitamins was associated with increased agreement with belief in the importance of taking vitamins (OR 1.91, 95% CI 1.08-3.34) [18].

While there is evidence on the impact of mobile phone messaging on health behavior change, little is known about the characteristics of effective programs and how to successfully implement such programs at a population level. In a 2010-2011 study, an evaluation of Text4baby assessing enrollment and usage among a prospective cohort of Atlanta, Georgia, women observed that despite high interest in the program, women who were younger (<26 years), less educated, and had lower literacy were more likely to have interrupted messages [19]. These findings indicate the importance of understanding patient perspectives on the feasibility of delivering SMS text message health promotion in underserved populations. Further, involving end users in all stages of text messaging program development, including piloting, implementation, and evaluation, allows developers to identify and address potential barriers to enrollment and use of the program [20]. This includes ensuring that the language used is lay friendly, culturally appropriate, and trauma informed to meet the needs of a broad target audience, including women experiencing conditions that contribute to vulnerability such as substance use and violence [9,21]. A 2015 Cochrane Review of the effects of mobile phone messaging interventions in health observed that pilot testing can assist in tailoring the messages to the preferences and needs of the target population [2].

In Canada, the use of mobile devices continues to increase [22] and creates an opportunity to reach a broad population base with health information. The vast majority of Canadian households (84.9%) subscribe to mobile phones and more Canadian households with incomes lower than average (<Can $51,804) are mobile phone-only households, suggesting that access and affordability drive Canadians’ communication usage [22]. A Pew Research Center survey conducted in 2015 with 1003 adult Canadians found that 63% of women of all ages and 94% of men and women aged 18-34 owned an advanced-feature mobile phone [23]. To ensure the relevancy and acceptability of using this mobile platform for prenatal education, we engaged Canadian women in the development of a prenatal education program delivered via one-way text message called SmartMom. We developed a prototype version of SmartMom, initially based on the delivery of Text4baby, which sends three evidence-informed messages per week to enrolled American women, timed to be relevant to their gestational age. The objective of this study was to explore women’s perceptions of the SmartMom program, paying particular attention to their perceptions about the message content, acceptability of receiving information by text message, positive health behaviors they might engage in after receiving a message, modifiable program factors, and intention to use the program.

## Methods

### The Intervention

A multidisciplinary group of researchers, clinicians, and allied health professionals initially adapted the message content from existing recognized public health resources, including British Columbia’s Baby’s Best Chance handbook [24], the Healthy Families BC website [25], and the British Columbia Maternity Care Pathway, a guideline for best practice for routine prenatal care in the province [26]. The experts engaged in multiple, iterative rounds of reviewing, revising, and weighting the messages. Research team members (SM, AH) edited the prototype messages for plain language and to ensure that each included an evidence-based behavior change strategy (ie, a physical activity message was supported by a goal-setting exercise). Messages were reviewed and endorsed by the Society of Obstetricians and Gynaecologists of Canada. The prototype SmartMom program consisted of 109 brief SMS text messages that focused on encouraging positive health behaviors, including increasing pregnancy- and childbirth-related knowledge, accessing routine antenatal assessments and recommended screenings, and adopting lifestyle behaviors to support healthy pregnancy and physiologic birth.

**Figure 1:**
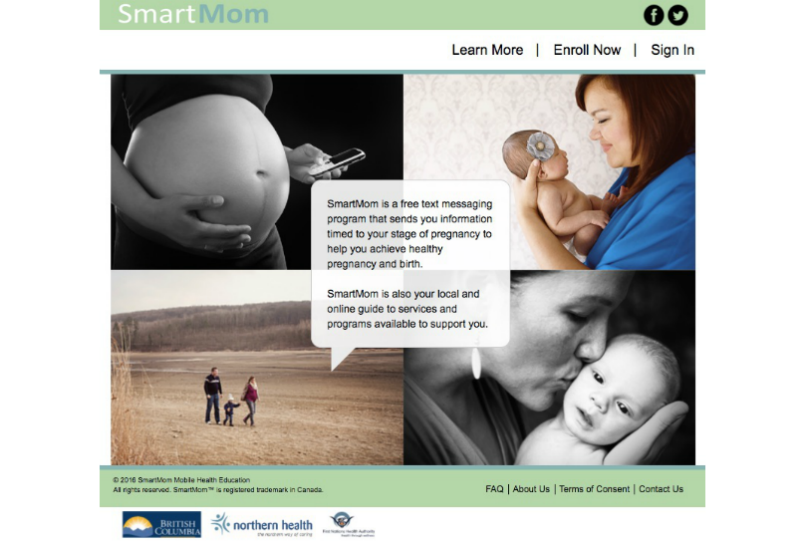
Home page for the SmartMom website.

**Figure 2:**
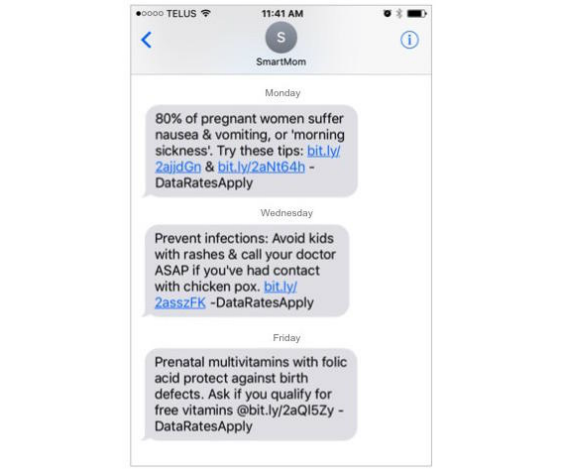
Example health promotion messages provided through the SmartMom program.

We propose that women who enroll in SmartMom will receive three SMS text messages per week, keyed to their gestational age beginning as early as 5 weeks gestation until their delivery. The communication will be one way and all women will receive the same messages for their region. Women and providers referring women to the program will have access to all messages on the program website [27]. Messages will be tailored to women’s gestational age and local region. Figure 1 presents the home page for the website. While the SmartMom program will be provided free of charge to women, data charges for browsing websites will not be covered. See Figure 2 for a sample of the messages.

### Settings and Participants

We conducted seven focus groups in three British Columbia communities in the Northern Health Authority (NHA), the region where the SmartMom pilot testing would take place. The NHA is the largest health region in the province, covering over two-thirds of British Columbia, and has on average 3500 births per year [28]. The behavioral research ethics boards of the University of British Columbia and the NHA provided ethical approval for the focus groups.

Participants were recruited through a range of methods: (1) third-party recruitment by public health nurses; (2) passive recruitment using study posters in community settings frequented by pregnant women and new mothers; and (3) passive recruitment through posting of study posters to Facebook groups involving childbirth, parenting, and community events for each study community. Recruitment via Facebook provided the greatest number of potential participants.

After contacting the research team, interested participants received an information letter outlining the purpose of the study and what their involvement would entail. Participants were then screened for eligibility; at the outset of each focus group, participants read and signed a study consent form that reflected the information letter. To participate, participants had to be English speaking, reside in the NHA, and be pregnant or have given birth in the past 12 months.

To minimize any barriers to participation for new mothers and women living in rural and remote communities, reimbursement was made available for childcare costs and travel expenses. Data collection was conducted in local settings that were convenient and familiar to new mothers, including public health units and family outreach centers. These settings had play spaces for children while their mothers participated in the focus group sessions. Participants received a Can $25 honorarium for their participation.

### Data Collection

In the first segment of the focus group, participants completed a demographic questionnaire, including four questions on mobile phone usage. Then a research team member (SM, AH) with experience in qualitative data collection guided a discussion on the women’s current information-seeking behavior. In the second segment, participants reviewed a printed copy of the SmartMom text messages and participated in a second moderated discussion, in this case on perceptions of the usability of the text message program. Open-ended questions explored participants’ perceptions about the message content, receiving information by text message, behavior they might engage in after receiving a message, modifiable program factors, and intention to use the program. Each focus group discussion was audiotaped and later transcribed verbatim for analysis.

### Data Analysis

Following thematic analysis [29] techniques, two members of the research team (SM, AH) first read and reread the focus group transcripts and then collaboratively developed an initial codebook of preliminary themes related to women’s perceptions of SmartMom’s strengths, weaknesses, and usability. Codes that expressed similar perceptions were grouped into categories. Constant comparison of emerging categories between transcripts helped to identify patterns and relationships in focus group discussions. The evolving codebook was refined into core categories and then tested for fit and relevance by two members of the research team (EG, NT). Using this focused codebook, the two coders (EG, NT) independently coded a sample of focus group transcripts. There were minimal disagreements in their interpretation of the transcripts. Discrepancies in interpretation were resolved through discussion with a third member of the research team (SM), who then entered the transcripts into NVivo for Mac version 10.1.3 (QSR International) qualitative data management software for organization. The thematic findings were then written into an explanatory narrative while two team members (AH, PAJ) led modifications to the SmartMom program based on the focus group feedback.

## Results

### Overview

Table 1 summarizes the demographic characteristics of the 40 women who participated in six focus groups in three rural northern communities. Among participants who had given birth, the rate of primary cesarean section was 19% (7/36). The majority of multiparous participants had received antenatal care from a family doctor (22/36, 61%), while the remainder received obstetrician (6/36, 17%) or midwifery (7/36, 19%) care, and one reported no birth attendant.

Table 2 describes the participants’ mobile phone usage. The vast majority of participants reported using a mobile phone (39/40, 98%), using text messages “all the time” (28/40, 70%), and surfing the Internet on their phone (37/40, 93%).

**Table 1:** Characteristics of participants (N=40).

Characteristic		n (%)
**Maternal place of birth**		
	Canada	37 (93)
	Mexico	1 (3)
	The United States	2 (5)
**Maternal age in years**		
	<20	3 (8)
	20-29	19 (48)
	30-39	18 (45)
**Lives with a partner**		
	Yes	32 (80)
	No	8 (20)
**Number of children given birth to**		
	0	3 (8)
	1	17 (43)
	2	12 (30)
	3	3 (8)
	4	3 (8)
	5	1 (3)
**Household annual revenue (Can $)**		
	<$35,000	14 (35)
	$35,000-$75,000	6 (15)
	>$75,000	15 (38)
	Prefer not to answer	5 (13)
**Highest level of education completed**		
	Primary school	1 (3)
	Graduated high school	11 (28)
	College or technical/trade program	13 (33)
	University degree	15 (38)

**Table 2:** Mobile phone usage survey (N=40).

Question from survey		n (%)
**Do you use a mobile phone?**		
	Yes	39 (98)
	No	1 (3)
**Do you use text messages?**		
	Yes	38 (95)
	No	2 (5)
**How often do you text people?**		
	All the time	28 (70)
	Occasionally	10 (25)
	Never	2 (5)
**Do you surf the Internet on your phone?**		
	Yes	37 (93)
	No	3 (8)

### Characteristics of the SmartMom Program

#### Enhances Knowledge of Options

Participants believed that the SmartMom messages enhanced their knowledge of pregnancy health-promotion strategies, particularly for first-time mothers. They also expressed how SmartMom gave them strategies to be active participants in their care:

I think that a lot of women, especially marginalized women, don’t really know that they have a choice. They don’t know what's coming up at the appointments and [SmartMom] might give them a heads up to ask those questions, when otherwise they wouldn’t know about it.

Focus Group (FG) 1

Participants believed that SmartMom would provide particular support to women in rural and remote communities where there are shortages of physicians, as the program would inform women of “different resources and other options” like public health programs (FG7).

#### Provides Knowledge in a Convenient and Timely Manner

A text message alert was perceived to be convenient for its immediacy and for being a free service:

I just like having the resource in my hands. You know, like right there connecting to a hyperlink.

FG7

Receiving messages three times per week and timed to gestational age was considered more ideal than the frequency of messages from existing apps or email newsletters. However, a number of participants described the transience of text message notifications as a potential barrier to use:

It’s a link buried in my text messages that I will never look at again.

FG6

#### Reflects How Women Access Information

Participants described text messaging as a ubiquitous mode of communication—“everyone texts” (FG5)—that reflects how women of their generation currently access pregnancy-related information via mobile phone data and the Internet. Participants indicated that having a text message was like a “bookmark,” and they could access a hyperlink later using free Wi-Fi (FG7). Only one participant in the sample had a phone without Internet access and was unable to access the hyperlinks.

#### Provides Support and Encouragement

The positive tone of the text messages was perceived to be “encouraging” and relatable and increased women’s interest to subscribe to the program. Participants expressed that harm reduction messages for drug and alcohol use (eg, encouraging women to reduce the negative effects of use) were confidence boosting and “not fear based” or “shaming” as some had found with prenatal resources. The overall tone of the messages was perceived to be “straightforward” and not “sugar coated.” One woman described SmartMom as a character:

She’s not your girlfriend. You wouldn't go out for coffee...I don't think she’d be, like, my friend, but I think she’s credible.

FG5

#### Reflects Local Knowledge and Resources

Participants expressed appreciation that SmartMom provided local, not national or international, information tailored to their region. Local content enhanced the credibility of the program and would increase participants’ likelihood of using SmartMom:

[SmartMom] is a reliable source and you can trust that. When you go to Google, you can find anything. It [the Internet] is very scary sometimes. But if you have a reputable text and you know where it’s coming from and you trust the source, then you’re more likely to go to that resource and believe it and seek it out.

FG3

#### Stimulates Behavior Change

The primary action that participants might take after reading a message would be to discuss it with their care provider or prepare a list of questions to ask:

I like that the one text was about making a list [of questions]. Because when you do have your prenatal appointments you maybe would feel a bit more prepared with some questions, things that had been brought up in the text messages. As opposed to trying to think of all the things that you want to ask your doctor at your one appointment. Because it’s not easy to just go in and ask a question, right?

FG6

A number of participants expressed that they may forget to bring up a topic because of the time gap between receiving a message and going to an antenatal visit. As one noted, “You get a text message, two or three weeks go by, you might not still be thinking about it at your appointment” (FG6).

Participants indicated the hyperlinks were the strongest feature of the SmartMom program:

I just like the links leading you to one of the articles or something. I was like “Oh I’d love to read that,” even open [the webpage] and even go back to it.

FG1

Phone numbers, in contrast, were perceived to be less useful than hyperlinks because they would not be saved in the same manner:

I would click on the link rather than call somebody. It’s just boom, and then you’re done. You just click it...I've got kids at home and they yell and scream.

FG1

SmartMom also includes interactive components intended to stimulate behavior change, such as knowledge quizzes, nutrition calculators, and links to interactive maps of local health services. Although participants did not have an opportunity to test these individual components, they welcomed the idea and felt these interactive components would help them stay engaged in the program.

### Modifiable Characteristics

To improve the SmartMom program further, participants also identified a range of modifiable characteristics.

#### Frequency and Timing

Certain topics were considered very important for the first weeks of pregnancy, particularly regarding accessing services:

Some of those moms are 14, 15, 16 years old and those are the ones that don’t have great information or even the knowledge how to access information. So getting it early to them would be good.

FG6

Some participants suggested receiving a “bundle” of messages upon enrollment related to prenatal classes, doula care and midwifery, and morning sickness triggers. Participants also wished to access the text messages outside of their SMS program to help them remember what to discuss with their care provider and what hyperlinked articles to read again. In response, we modified the program to include key messages earlier in pregnancy and provided a full list of the messages on the SmartMom website. At the participants’ suggestion, we also removed any repetition of messages related to drugs and alcohol.

#### Relevance

Some messages were perceived to be relevant for some women and not for others. Some participants suggested that they would disenroll from SmartMom if the harm reduction messages were repetitive, irrelevant, or countered their beliefs:

Could you have like a questionnaire in the beginning where you’re like, “I’m not wanting information on this, this, and this”?... If I was being shown information that I didn’t need, I wouldn’t want to be a part of [SmartMom].

FG4

To address this concern, we changed the program to provide women with one message on each topic (eg, smoking), which will be accompanied by an option to receive more messages on that topic. We also developed *supplemental streams* of messages tailored to individual pregnancy needs: (1) smoking, (2) alcohol, (3) depression, (4) pregnancy after previous cesarean, (5) obesity, (6) pregnancy loss, (7) new immigrants, (8) >35 years of age, and (9) exposure to violence. Women can opt in to receive these streams at the time of enrollment.

#### Support and Affirmations

Participants felt that the SmartMom prototype did not meet their needs for social and emotional support and support from peers:

It would be so cool if there could be like a [local] forum for women. Like if you’re connected by your postal code then there would be an actual online community of women in your area that you could actually meet up with.

FG1

We consequently added hyperlinks that listed local pregnancy and parenting groups for users to have face-to-face interaction with other women. We also enhanced the supportive language and tone of the messages by adding more encouraging messages or *affirmations* —“You’re doing great!”—which participants believed would keep them motivated, maintain their interest in receiving future texts, and enhance their confidence in making healthy choices.

#### Enrollment

In our original protocol for the implementation of SmartMom, we proposed that potential users would enroll via an existing, trusted information source (eg, doctor, public health nurse, or British Columbia Ministry of Health website). However, participants noted that women in their region commonly do not attend their first prenatal appointment until their second trimester. They reflected that, instead, women would be easiest to reach in early pregnancy via social media:

I think here social media campaigning is a great way to get the word out. There seems to be huge groups on Facebook that have thousands of women in them. That would be a great way to get it out, at least in this town.

FG6

To address this, we expanded our enrollment plan to advertise SmartMom via Facebook, other social media platforms, and in the pregnancy test section of pharmacies.

#### Postpartum

Participants strongly recommended extending the SmartMom program of messages for the postpartum period and first year of newborn life. They expressed that there were few timely, evidence-based resources readily available to meet their postpartum needs, as stated by a participant in one group:

At the end of pregnancy and after you’ve had your baby, resources for that as well. If there’s a way to say at the end, “I've had my baby, now start sending me the other one.” Because those first few weeks are very crucial, very hard. Very, very hard.

FG1

In response, we developed prototype SmartMom messages for birth to age one, which will be evaluated in a separate study.

## Discussion

### Principal Findings

The results of our thematic analysis indicate that participants perceived SmartMom to be highly acceptable and relevant for childbearing Canadian women. Our findings suggest that SmartMom will enhance women’s knowledge of health promotion strategies in a convenient and timely manner, provide support, and encourage women to engage in new behavior, such as discussing the text message topics with their care provider at the appropriate gestational age. The text message modality reflected how participants currently sought pregnancy-related information and provided them with local information tailored to their gestational age, which they had not received through other pregnancy resources. Eliciting the perspectives of potential SmartMom users early in the design process resulted in the identification of factors that allowed us to modify and tailor the program to address the perceived needs of childbearing women in the study region. Key modifications included creating supplemental streams of messages for subgroups of women (ie, smoking and pregnancy after previous cesarean). We also added the entire suite of messages to the SmartMom website and provided hyperlinks to local programs that provide peer-to-peer social support in pregnancy.

The SmartMom program is novel in that it is tailored to offer the right prenatal health message at the right time to the right woman. Text messages serve as a cue to action with salient information for pregnant women, essentially providing just-in-time, locally relevant tips and resources to help women make healthy prenatal and postpartum health choices based on their individual needs [30]. To our knowledge, SmartMom is the first peer-reviewed, evidence-based mHealth program for prenatal education in Canada and the first to be endorsed by the Society of Obstetricians and Gynaecologists of Canada, the professional organization that establishes evidence-based standards of maternity care in Canada. Previous tools have been developed for the US context and women in this study cited using them. O’Donnell and colleagues compared the content of two free mobile phone apps for US women, Text4baby and BabyCenter’s *My Pregnancy Today*, and determined that <20% of the messages delivered by either program contained information that explicitly addressed recommended prenatal care content [31]. The health care product company Johnson & Johnson owns BabyCenter, which also provides *My Pregnancy Today* to Canadian populations, but no studies have been published on the development of that app. In contrast, we explicitly sought to develop SmartMom based on information from existing peer-reviewed, evidence-based Canadian resources [24-26].

The Text4baby program underwent iterative cycles of user testing with childbearing women prior to its launch in 2010 [16]. In a commentary on the Text4baby development, Whitaker et al indicated that the testing included the following: (1) informal discussion groups with women from community health centers in six cities across the United States regarding the message topics and frequency; (2) health literacy testing of the messages with 100 pregnant African American, low-income women in the Women, Infants, and Children program waiting room at a large city hospital in Atlanta, Georgia; and (3) beta testing of the program with a sample of 10 childbearing women [16]. However, it is unclear what demographics of women were involved, what methods were used for data collection and analysis, and what findings emerged from the user testing.

Recent qualitative studies on women’s views of mHealth pregnancy interventions conducted in Argentina and Australia found, similar to our study, that exposure would enhance women’s perceived access to high-quality, pregnancy-related information [32], but would not meet women’s needs for two-way interaction [33]. To address this gap, we included hyperlinks to local pregnancy and parenting groups for women to interact with peers and health care professionals. Future iterations of the program may benefit from further interactive components.

In our study, we conducted a qualitative investigation with childbearing women to ensure that SmartMom “will reflect an understanding of context, of the users, of the functionality of the system (and thus the users’ needs), and of the software development process” [34]. We used feedback from women to modify the program before conducting pilot testing on a larger scale. This aimed to ensure that the program will be usable and acceptable and to avoid any program errors that would threaten our stakeholder partners’ investment in the program. Findings from our analysis may be transferable to prospective SmartMom user populations of childbearing women in similar geographic and health service settings in Canada.

### Limitations

It is possible that the perspectives of some end users may not have been captured in this study. Participants were recruited primarily via Facebook and, consequently, perspectives may be overrepresented by women who have Internet and/or mobile phone access. We did not purposefully sample for Aboriginal and First Nations women or report separately on the unique perspectives of this population group. In addition, we did not purposefully recruit women from communities with no mobile phone coverage or women with no local maternity services. The perspectives of these women will be elicited through focus groups during the next phase of the SmartMom development—pilot usability testing—which will include summative evaluation through user surveys and further focus groups.

### Strengths

This study was strengthened by the diverse sample of childbearing women. This provided a rich understanding of women’s perceptions of the SmartMom program and the context in which they will use it. Our inductive, team-based analysis enhanced the rigor and credibility of findings. A final strength of the study was our testing of a prototype *wireframe* version of the SmartMom program prior to developing the text message software and investing in an expensive technology platform. The usability and acceptability findings from this study will inform the next phase of the research process, which includes testing the messages in a real-world pilot feasibility study where rural pregnant women enroll in the SmartMom program and interact with the messages and website in real time.

### Conclusions

This formative qualitative evaluation provides evidence that SmartMom is acceptable to end users. Exploring the perspectives of childbearing women in British Columbia resulted in the tailoring and modification of the program to their specific needs, which we anticipate will enhance SmartMom’s usability. These findings support the initiation of a pilot study to investigate the usability of the SmartMom program at a population level and explore the effects of the messages on adoption of health-promoting behaviors and maternal-child health outcomes.

## Abbreviations

FG: focus group
NHA: Northern Health Authority
OR: odds ratio
SMS: short message service
